# Investigation on Threshold Voltage Adjustment of Threshold Switching Devices with HfO_2_/Al_2_O_3_ Superlattice on Transparent ITO/Glass Substrate

**DOI:** 10.3390/mi11050525

**Published:** 2020-05-21

**Authors:** Yejoo Choi, Jaemin Shin, Seungjun Moon, Changhwan Shin

**Affiliations:** Department of Electrical and Computer Engineering, Sungkyunkwan University, Suwon 16419, Korea; mirmail9206@skku.edu (Y.C.); spacewhap@skku.edu (J.S.); scott93@skku.edu (S.M.)

**Keywords:** transparent device, indium tin oxide (ITO), threshold switching device, multilayer, non-volatile memory

## Abstract

Threshold voltage adjustment in threshold switching (TS) devices with HfO_2_/Al_2_O_3_ superlattice (by means of changing the cycle ratio of HfO_2_ to Al_2_O_3_ in atomic layer deposition) is investigated to implement a transparent cross-point array. TS devices with different cycle ratios (i.e., 3:1, 3:2, and 3:3) were fabricated and studied. The threshold voltage of the devices was increased from 0.9 V to 3.2 V, as the relative contents of Al_2_O_3_ layer in the superlattice were increased. At the same time, it is demonstrated that the off-resistance values of the devices were enhanced from 2.6 × 10^9^ to 6 × 10^10^ Ω as the atomic layer deposition (ALD) cycle ratio of HfO_2_ to Al_2_O_3_ layer was adjusted from 3:1 to 3:3. However, the hold voltage and the on-current values were almost identical for the three devices. These results can be understood using the larger barrier height of Al_2_O_3_ layer than that of HfO_2_ layer.

## 1. Introduction

Transparent electronics have been ardently investigated in a variety of areas such as bioelectronics, environmental-engineering, display engineering, and wearable electronics as replacements and/or substitutions for conventional opaque electronics, because the optical transparency leads to functional merits in various applications [1,2,3,4,5]. For example, opaque electrodes (e.g., platinum) have been used as implantable neural probes for brain-machine interfaces. The opaque electrodes hamper observations on the section under the electrodes. For this reason, transparent electrodes such as graphene have been investigated as the candidate for implantable neural probes for brain-machine interfaces. Among those transparent devices, transparent non-volatile memory devices (e.g., resistive random-access-memory (RRAM) devices) have drawn growing attention for use in transparent display panel and circuitry, because non-volatile memory devices have (i) a simple two terminal structure (i.e., top electrode-dielectric layer-bottom electrode), (ii) a high power efficiency (i.e., low power consumption), and (iii) a 4F^2^ cell size [6,7,8,9,10,11,12,13]. However, cross-point array using non-volatile memory devices has the technical issue of its sneak current path, due to the low off resistance of non-volatile memory devices. This undesirable sneak current can arise from nearby-unselected cells when non-volatile memory devices are configured as an array. This would hurt the read margin of non-volatile memory devices, as well as limit the maximum size of the crossbar array [14,15]. For alleviating those issues, a volatile resistive switching device called a threshold-switching (TS) device has been studied [16,17,18]. When a TS device is connected to a non-volatile memory device in series, the device structure can show non-volatile memory characteristics, avoiding unexpected sneak leakage current [19]. Although investigations on TS devices have been widely implemented for addressing these issues, studies on transparent TS devices are lacking (even though studies on transparent non-volatile memory devices are available).

Moreover, among various TS devices, metal ion TS devices (which show abrupt resistive switching through the forming/rupture of metal ion filaments) have been highlighted because they can have a high on/off ratio (≥10^6^) and fast switching speeds (≤100 ns) [20,21,22,23,24]. Because of those outstanding electrical properties, TS devices have been utilized for other applications, such as phase-transition field effect transistor (FET) as well as cross-point arrays. From this point of view, the operational reliability of TS devices needs to be improved for being used in various fields. Therefore, various reliability issues such as endurance, cycling stability, temperature stability, etc. have been studied. For example, the operational failure in a TS device mainly originates from the diffusion of active metal into the oxide layer. For barricading the diffusion, a TiN diffusion barrier is usually inserted between the active metal electrode (i.e., top electrode) and the oxide layer [25]. By adding the diffusion barrier, the diffusion can be restricted, and thus the device can show better endurance characteristics. Moreover, a large area of electrodes lets a large number of filaments be formed in the oxide layer. In other words, there are many paths for current flow. This results in variation between operations (i.e., cycle-to-cycle variation). This issue can be resolved by decreasing the device area [26,27]. As the device area is scaled-down, the number of filaments in the oxide layer is decreased, resulting in a decrease in cycle-to-cycle variation. Furthermore, active metal alloy with chalcogenide material (i.e., AgTe) was used as metal electrode for increasing the endurance and switching speed, because the chalcogenide material contributes to the fast dissolution of conductive filament. Moreover, investigations on threshold voltage adjustment have been performed. This is because, for sufficiently suppressing the sneak current, the threshold voltage of a TS device should be designed in-between the half-set voltage and the set voltage of a non-volatile memory device [19]. In a cross-point array, the half-set voltage is applied to half-accessed cells. Thus, if the threshold voltage of a TS device is less than the half-set voltage, the cell cannot benefit from the high off-resistance of the TS device, resulting in a sneak current. Furthermore, when the threshold voltage is higher than the set voltage, the current becomes restricted by the high off-resistance of the TS device, even if the set voltage is applied to the non-volatile memory device. For balancing those two voltage values, various methods (e.g., adding a diffusion barrier on a dielectric layer and/or changing a bottom electrode material) to adjust the threshold voltage of metal ion TS devices have been studied [22,23,24,25,28]. However, these methods are hardly applied for transparent applications because (i) the number of practical transparent substrates is very limited and (ii) adding a new layer can aggravate the transparency. Thus, in this study, by varying the atomic layer deposition (ALD) cycle ratio of HfO_2_ and Al_2_O_3_ for HfO_2_/Al_2_O_3_ superlattice (HAO), the threshold voltage adjustment of TS devices on transparent indium tin oxide (ITO)/glass substrate is demonstrated for realizing transparent cross-point array. HfO_2_ and Al_2_O_3_ layer are well-known as transparent dielectric layers, and thus do not adversely affect the transparency. The threshold voltage was increased from 0.9 to 3.2 V as the ratio of HfO_2_ to Al_2_O_3_ in HAO was changed from 3:1 to 3:3. Furthermore, as the cycle ratio was adjusted from 3:1 to 3:3, the off resistance at 0.5 V was improved from 2.6 × 10^9^ to 6 × 10^10^ Ω. However, it is demonstrated that the hold voltage and on-resistance are not affected by the presence of Al_2_O_3_ layers. These measurement results should be originated from suppressing the diffusion of top electrode (i.e., silver) by inserting an Al_2_O_3_ layer into HfO_2_ layer. We expect that this experiment would help to expediate the development of a transparent cross-point array in the near future.

## 2. Fabrication and Measurement

**Figure 1:**
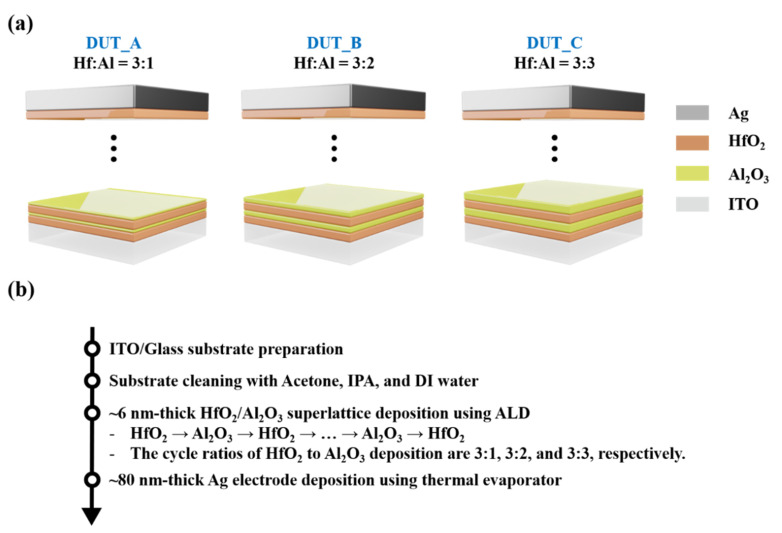
(**a**) Structure of HfO_2_/Al_2_O_3_ superlattice (HAO)-based TS devices: DUT (Device Under Test)_A (left), DUT_B (middle), and DUT_C (right). (**b**) Fabrication process of HAO-based TS devices on a transparent ITO/glass substrate.

A HAO-based TS device was fabricated as follows: First, an ITO-coated glass substrate (AMG, Uiwang, Korea) was prepared as a bottom electrode. ITO has been widely utilized as transparent substrate due to its high transparency (i.e., near 90% in the visible wavelength range) and low resistivity (i.e., ~10^−3^ Ω⋅cm). The substrate was dipped/cleaned in acetone (Samchun Chemicals, Pyeongtaek, Korea), isopropyl alcohol (IPA) (Daejung Chemicals & Metals Co., Siheung, Korea), and deionized water (DI water), in order, using sonicator (Sungdong Ultrasonic Co., Seoul, Korea) each 5 min. After the cleaning process, the HAO film was deposited using ALD (NCD Lucida-D100, NCD, Daejeon, South Korea) at 200 °C of chamber temperature. The chamber pressure was maintained below 5 × 10^−2^ torr. The cycle ratio of HfO_2_ to Al_2_O_3_ layer is 3:1, 3:2, and 3:3 for DUT (Device Under Test)_A, DUT_B, and DUT_C, respectively. Subsequently, 3 cycles for HfO_2_ were deposited on the superlattice, keeping the vacuum condition for equalizing the interface material (i.e., HfO_2_) to the top electrode and bottom electrode. The deposition rate of HfO_2_ and Al_2_O_3_ is ~0.85 and ~1.1 Å/cycle, respectively. Tetrakis(ethylmethylamino) Hafnium (TEMAHf) and Trimethylaluminum (TMA) were used as precursors for depositing HfO_2_ and Al_2_O_3_ layer, respectively. The temperature of TEMAHf was maintained at ~80 °C, and the temperature of TMA was maintained at ~10 °C. The total thickness of the HAO layer is 6 nm. In this work, any post-annealing processes were not implemented. So, the HAO layer does not show ferroelectricity because it was not crystallized to orthorhombic phase which shows ferroelectricity due to the lack of annealing temperature (note that ferroelectric HAO layer is usually crystallized at 700–900 °C) [29,30]. Lastly, 80-nm-thick Ag top electrodes were deposited by means of a thermal evaporator (Korea Vacuum KVETE-T4560, Korea Vacuum Limited, Daegu, Korea) and patterned using shadow mask. The active areas of the fabricated devices are 3.14 × 10^−4^ cm^2^. The structure and fabrication process are described in Figure 1. The electrical characteristics of the devices were measured using a Keithley 4200A-SCS (Keithley, Cleveland, OH, USA) semiconductor parameter analyzer at 300 K.

## 3. Results and Discussion

**Figure 2:**
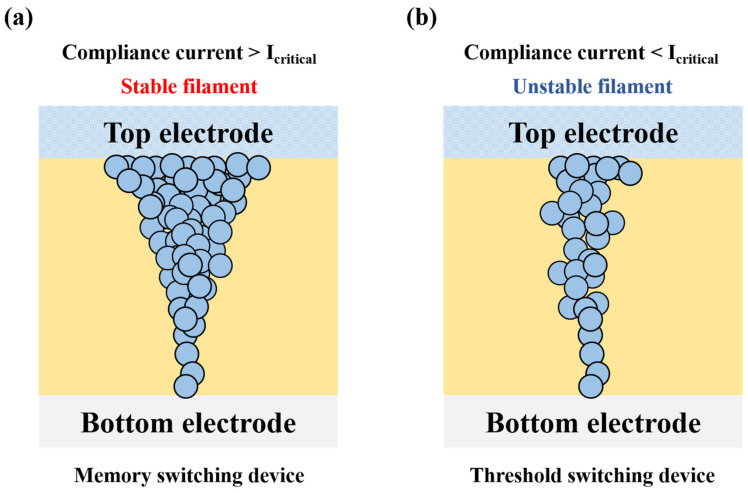
(**a**) Illustration of filaments in memory switching device. The stable filaments are formed in the dielectric layer with the compliance current higher than a critical current value. The filament is composed of top electrode material. (**b**) Illustration of filaments in threshold voltage switching device. The unstable filaments are formed in the dielectric layer with the compliance current smaller than a critical current value. Therefore, the unstable filament can be dissolved with a sufficiently small positive voltage (i.e., hold voltage). The filament is composed of top electrode material.

Metal ion resistive switching devices can show both non-volatile (i.e., memory switching) and volatile resistive switching (i.e., threshold switching) properties by means of the forming/rupture of metal ion filaments. After the initial forming process, when the maximum current flowing through a metal ion resistive switching device is higher than a critical value (*I_critical_*), stable filaments consisting of active metal are formed in the oxide layer at the set voltage. Note that the set voltage indicates a specific voltage forming metal ion filaments in a non-volatile resistive switching device. Afterwards, the stable filaments start to be ruptured when the reset voltage is applied to the device. Note that the reset voltage indicates a critical voltage, which dissolves metal ion filaments in a non-volatile resistive switching device. The reset voltage usually has the opposite polarity to the set voltage. In this case, the low resistance can be maintained even if a voltage is not applied to the device after the forming process, and thus the device has non-volatile memory switching properties (i.e., conductive-bridge random access memory). However, when the maximum current flowing through a metal ion resistive switching device is limited below the critical value, unstable filaments consisting of active metal are formed in the oxide layer at the threshold voltage. Contrary to the previous case (i.e., memory switching), in this case, the unstable filaments start to be ruptured at the hold voltage which has the same polarity as the threshold voltage. The reason for this phenomenon is that, as the maximum current flowing through the device is restricted, the filaments become easy to transform to active metal clusters (i.e., dissolution of the filaments). At the hold voltage (which has the same polarity as the threshold voltage), the filaments are ruptured into the active metal clusters, so that the resistance of the device is abruptly increased because the clusters do not make the top electrode (i.e., active metal) to be connected with the bottom electrode (i.e., inert metal). The threshold voltage and hold voltage indicate a critical voltage of triggering the resistive switching from a high resistance state to a low resistance state, and from a low resistance state to a high resistance state in a volatile resistive switching device (i.e., a TS device), respectively. Metal ion resistive switching devices, which show non-volatile (i.e., memory switching) and volatile resistive switching (i.e., threshold switching) characteristics, are illustrated in Figure 2 [31,32,33].

**Figure 3:**
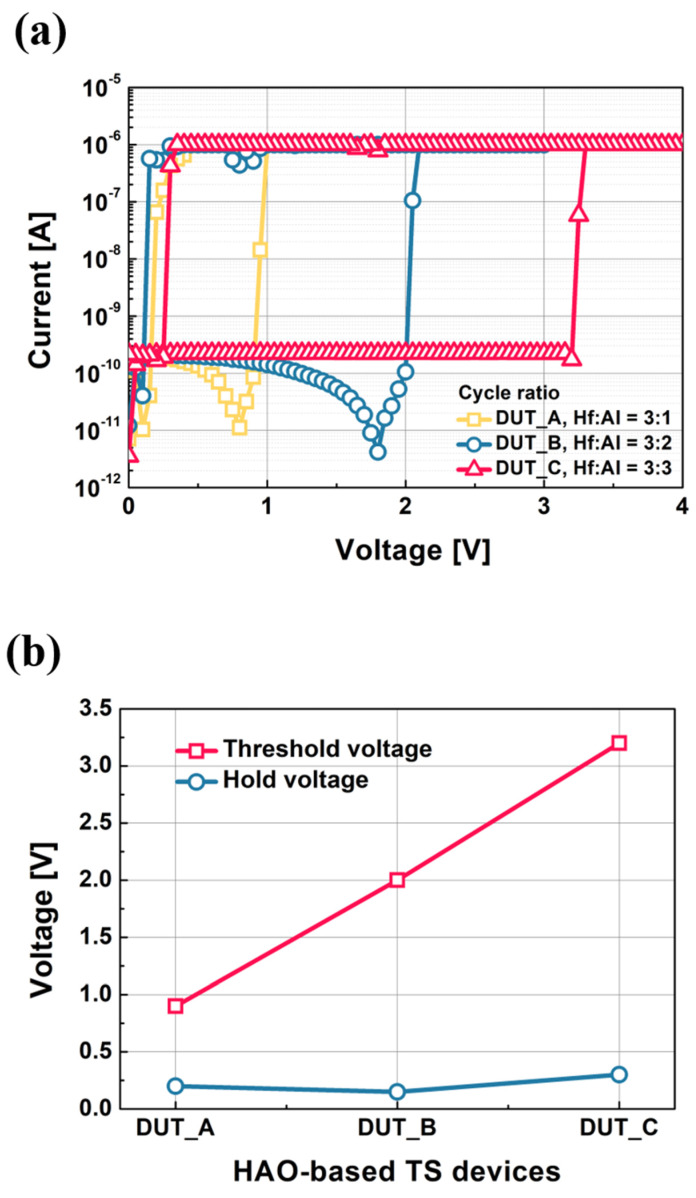
(**a**) Measured current vs. voltage of the HAO−based TS devices: DUT_A, DUT_B, and DUT_C. The compliance current is set to 10^−6^ A for realizing threshold switching characteristics. (**b**) The threshold voltage and hold voltage characteristics of the fabricated HAO-based TS devices. DUT_A, DUT_B, and DUT_C have a HfO_2_:Al_2_O_3_ cycle ratio of 3:1, 3:2, and 3:3, respectively.

In practical measurement, the maximum current can be modulated by setting the compliance current level. However, it is noteworthy that, in real electronic circuits, blocks for limiting the current flow through a device will take up an area. This area penalty must be solved for realizing future low power applications using metal ion TS devices. In this work, the compliance current is set to 10^−6^ A for realizing the threshold switching characteristics of the devices. Figure 3a shows the measured current vs. voltage characteristics of the fabricated HAO-based TS devices (i.e., DUT_A, DUT_B, and DUT_C). The devices show abrupt volatile resistive switching characteristics (i.e., threshold switching). Figure 3b plots the threshold voltage and hold voltage of three DUTs which have different HfO_2_:Al_2_O_3_ cycle ratios.

**Figure 4:**
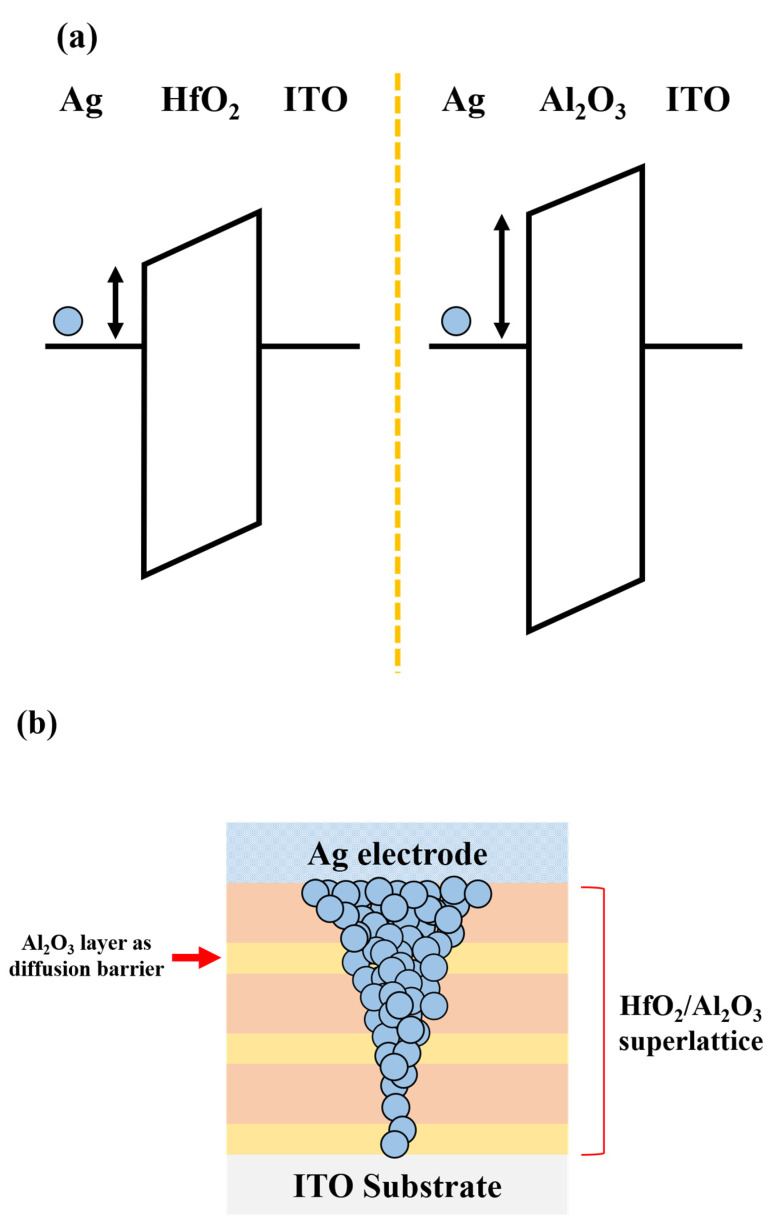
(**a**) The energy band diagram of Ag/HfO_2_/ITO device (**left**) and Ag/Al_2_O_3_/ITO device (**right**). Due to the larger bandgap energy (i.e., 7 eV) of Al_2_O_3_, the Al_2_O_3_ layer acts as diffusion barrier, and thus it increases the threshold voltage and the off-resistance value. (**b**) The cross-sectional view of HfO_2_/Al_2_O_3_ superlattice TS device. In this superlattice, the Al_2_O_3_ layers act as a diffusion barrier.

As the cycle for the Al_2_O_3_ layer is increased (i.e., from DUT_A to DUT_C, in order), the threshold voltage is increased from 0.9 to 3.2 V. This is because the bandgap energy (~7 eV) of Al_2_O_3_ is higher than the energy (~5.7 eV) of HfO_2_, and thus, as described in previous works, this wider bandgap works as the metal ion (i.e., Ag) injection barrier, as shown in Figure 4 [34]. Contrary to the case of threshold voltage, the hold voltages of the three DUTs are almost identical to each other. The hold voltages of DUT_A, DUT_B, and DUT_C are 0.2, 0.15, and 0.3 V, respectively. This indicates that the dissolution of filaments is not affected by the bandgap energy of dielectric layer under the condition of 1 μA of compliance current.

**Figure 5:**
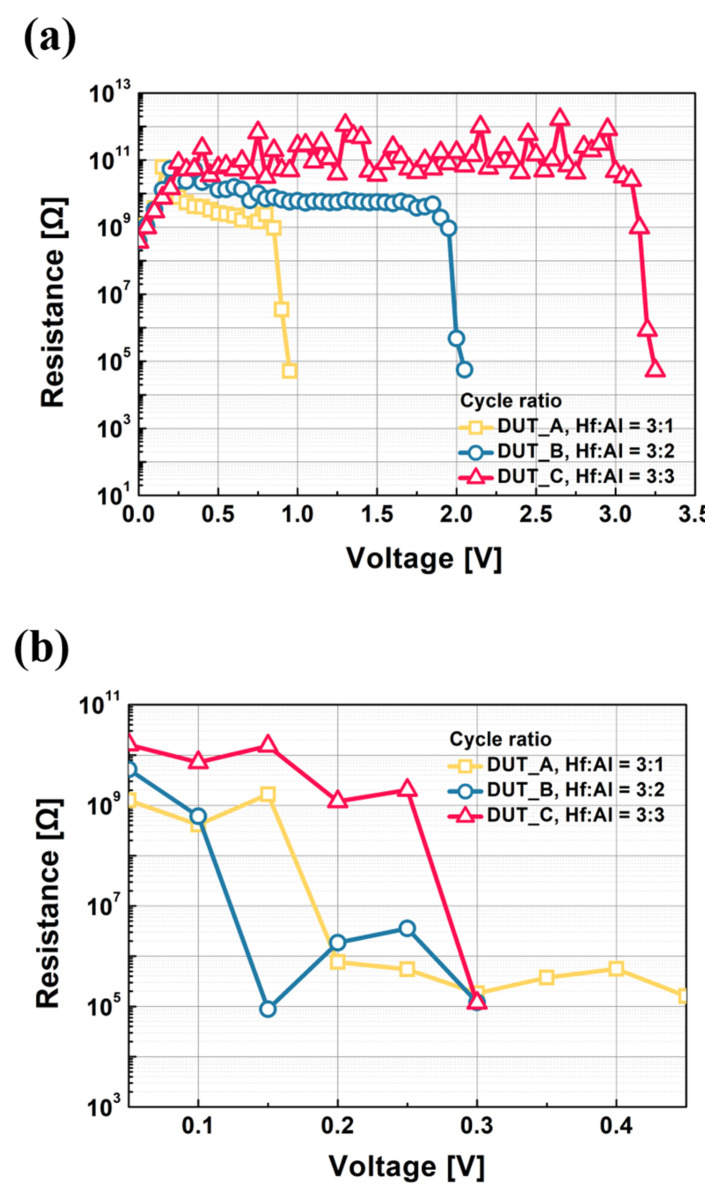
(**a**) The measured resistance vs. voltage of the HAO-based TS devices before being turned-on: DUT_A, DUT_B, and DUT_C. (**b**) The measured resistance vs. voltage of the HAO-based TS devices before being turned-off: DUT_A, DUT_B, and DUT_C. When the devices are fully turned-on, the resistance values are not extracted because the on-current values are identical to the compliance current of 10^−6^ A.

Figure 5a,b illustrate the measured resistance vs. voltage of the DUTs from a high resistance state to a low resistance state, and from a low resistance state to a high resistance state, respectively. The resistance values of fully turned-on devices are not extracted, because the current level is fixed to the compliance current of 10^−6^ A. The off-resistance values of the devices before forming filaments (i.e., from high resistance state to low resistance state) are increased from 2.6 × 10^9^ to 1.3 × 10^10^ to 5.9 × 10^10^ Ω at 0.5 V as the HfO_2_:Al_2_O_3_ cycle ratio is manipulated from 3:1 to 3:3 (i.e., from DUT_A to DUT_B, in order). On the other hand, the resistance values (i.e., 5 × 10^4^ Ω) before being fully turned-on are almost identical for all the three DUTs. Likewise, the off-resistance values after the rupture of filaments (i.e., from low resistance state to high resistance state) are increased from 1.26 × 10^9^ to 1.6 × 10^10^ Ω as the cycle ratio is adjusted from 3:1 to 3:3 (i.e., from DUT_A to DUT_B, in order). The resistance values before the rupture of filaments are almost identical to each other. These results are understood by the low diffusivity of Ag ions in the Al_2_O_3_ layer [35,36]. The high barrier of the Al_2_O_3_ layer hampers the diffusion of Ag ions in the dielectric layer, and hence the resistance is increased. However, in the case of on-resistance, the stability of the formed filament is affected by the compliance current level, regardless of the diffusivity of Ag ions. Therefore, the on-resistance values are almost the same for all the three DUTs under the condition of 1 μA of compliance current.

In this work, it is observed that the insertion of an Al_2_O_3_ layer into a HfO_2_ layer can adjust the threshold voltage as well as improve the off-resistance values. These results would help to balance the set voltage of a non-volatile memory device and the threshold voltage of a TS device for configurating a cross-point array. Besides, other applications using TS devices (e.g., phase-transition FET, etc.) could benefit from these results. For instance, the minimum drain voltage to turn-on phase-transition FETs is determined by the threshold voltage of TS devices. Therefore, this threshold voltage adjustment method helps to tune the threshold voltage of phase-transition FETs. Furthermore, this method has no adverse impacts on the transparency and the device thickness because it does not need an additional diffusion barrier of metal ions, such as TiN liner, and the HfO_2_ and Al_2_O_3_ layers are transparent. Therefore, this method is more appropriate for transparent applications than the method of adding a diffusion barrier layer when the threshold voltage needs to be adjusted. However, there is still room for more studies in future. First, the other impacts of tuning the threshold voltage should be investigated. As the threshold voltage is increased by means of the insertion of an Al_2_O_3_ layer, a higher voltage needs to be applied to the device for turning-on the device with the same film thickness. In other words, a stronger electric field needs to be applied to the device with an inserted Al_2_O_3_ layer. This means that the insertion of Al_2_O_3_ layer might adversely affect the reliability. Furthermore, the effects on the volatility need to be studied. In this work, 1 μA of the compliance current is used for realizing the threshold switching of the device. It is known that a HfO_2_-based TS device can show threshold switching properties at ~100 μA of compliance current [21]. This compliance current for inducing the threshold switching is decided by the difference between the energy of metal cluster and the energy of metal filament. Hence, the insertion of the Al_2_O_3_ layer could affect the energy difference, changing the maximum compliance current for causing the threshold switching. Finally, although transparent dielectric layers (i.e., HfO_2_ and Al_2_O_3_ layers) and bottom electrode (i.e., ITO/glass substrate) are used in this work, the devices are not fully transparent due to the top electrode (i.e., silver). It has been demonstrated, however, that the silver electrode can be transparent as its thickness becomes sufficiently thin (~10 nm), or if the electrode is composed of silver nanowire [37,38,39]. Moreover, it has recently been proposed that TS devices with a nickel top electrode can show a sufficiently high on-current (~1 mA) with threshold switching characteristics [21]. In addition, it has been demonstrated that nickel and gold alloy can be transparent by means of a rapid thermal annealing process. Therefore, as future works for fully transparent cross-point array, transparent TS devices with thin silver electrode, silver nanowire electrode, or transparent electrodes (e.g., Ni/Au) need to be investigated [40,41,42].

## 4. Conclusions

In this study, to suppress the sneak current path in a transparent non-volatile memory device cross-point array, the method of manipulating the threshold voltage of TS devices with HfO_2_/Al_2_O_3_ superlattice is explored. The ALD cycle ratio for the HfO_2_/Al_2_O_3_ superlattice is adjusted from 3:1 to 3:3 to modify the threshold voltage and off-resistance. As the cycle ratio is varied from 3:1 to 3:3, the threshold voltage increases from 0.9 to 3.2 V, due to the wider bandgap of Al_2_O_3_ (~7 eV) than that of HfO_2_ (~5.7 eV). On the other hand, there is no relationship between the cycle ratio and hold voltage. The off-resistance value of the devices is increased from 2.6 × 10^9^ to 6 × 10^10^ Ω when the cycle ratio is modified from 3:1 to 3:3 because of the low diffusivity of Ag ions in the Al_2_O_3_ layer. However, the on-resistance values are almost identical for the three DUTs, because the on-current is regulated by the compliance current of 10^−6^ A. Lastly—but not least—various future works for materializing fully transparent cross-point arrays with TS devices are proposed.
